# Bis(1,5-diphenyl­carbazonato)di­methano­lcobalt(II)

**DOI:** 10.1107/S1600536809055305

**Published:** 2010-01-09

**Authors:** Yanmei Chen, Bin Xu, Shixiong She, Bin Hu, Yahong Li

**Affiliations:** a Qinghai Institute of Salt Lakes, Chinese Academy of Sciences, Xining 810008, People’s Republic of China; b Key Laboratory of Organic Synthesis of Jiangsu Province, College of Chemistry and Chemical Engineering, Suzhou University, Suzhou 215123, People’s Republic of China

## Abstract

The structure of the title compound, [Co(C_13_H_11_N_4_O)_2_(CH_3_OH)_2_], is a mononuclear six-coordinated octa­hedral cobalt(II) complex of *C*_i_ mol­ecular symmetry. The Co^II^ ion is coordinated by two N atoms and two O atoms from two 1,5-biphenyl­carbazide ligands, and two O atoms from two methanol molecules. Two diphenyl­carbazidate ligands and the central Co^II^ ion form the basal plane, with the two methanol mol­ecules located in axial positions. The crystal packing is defined by bifurcated O—H⋯N hydrogen bonding and intra­molecular N—H⋯O inter­actions.

## Related literature

For the use of biphenyl­carbazide for the analytical determination of chromium in biological materials, see: Yarbro & Flaschka (1976 ▶). For its coordination modes, see: Feigl (1924 ▶); Shafranskii & Mal’kova (1975*a* ▶,*b* ▶); Martynova *et al.* (1985 ▶); Turkington & Tracy (1958 ▶); Deshpande & Jain (1988 ▶). For related literature, see: Pankaj & Chauhan (2004 ▶); Sollott & Peterson (1969 ▶); Cazeneuve (1900*a* ▶,*b* ▶).
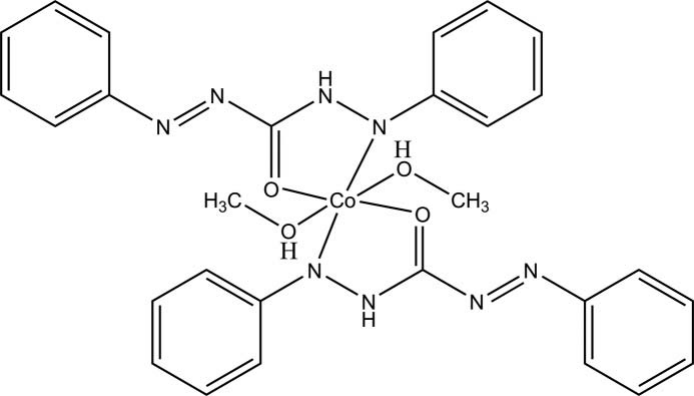


## Experimental

### 

#### Crystal data

[Co(C_13_H_11_N_4_O)_2_(CH_4_O)_2_]
*M* *_r_* = 601.53
Monoclinic, 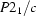

*a* = 6.492 (2) Å
*b* = 8.926 (3) Å
*c* = 25.159 (9) Å
β = 92.372 (6)°
*V* = 1456.7 (9) Å^3^
*Z* = 2
Mo *K*α radiation
μ = 0.64 mm^−1^
*T* = 296 K
0.35 × 0.28 × 0.27 mm

#### Data collection

Bruker SMART CCD area-detector diffractometer
Absorption correction: multi-scan (*SADABS*; Bruker, 2001 ▶) *T*_min_ = 0.808, *T*_max_ = 0.847
6990 measured reflections
2564 independent reflections
2037 reflections with *I* > 2σ(*I*)
*R*_int_ = 0.046

#### Refinement

*R*[*F* ^2^ > 2σ(*F* ^2^)] = 0.042
*wR*(*F* ^2^) = 0.117
*S* = 1.08
2564 reflections
196 parameters
H atoms treated by a mixture of independent and constrained refinement
Δρ_max_ = 0.40 e Å^−3^
Δρ_min_ = −0.43 e Å^−3^

### 

Data collection: *SMART* (Bruker, 2001 ▶); cell refinement: *SAINT* (Bruker, 2001 ▶); data reduction: *SAINT* program(s) used to solve structure: *SHELXS97* (Sheldrick, 2008 ▶); program(s) used to refine structure: *SHELXL97* (Sheldrick, 2008 ▶); molecular graphics: *SHELXTL* (Sheldrick, 2008 ▶); software used to prepare material for publication: *SHELXTL*.

## Supplementary Material

Crystal structure: contains datablocks I, global. DOI: 10.1107/S1600536809055305/kp2244sup1.cif

Structure factors: contains datablocks I. DOI: 10.1107/S1600536809055305/kp2244Isup2.hkl

Additional supplementary materials:  crystallographic information; 3D view; checkCIF report

## Figures and Tables

**Table 1:** Selected bond lengths (Å)

Co1—O1	2.0263 (18)
Co1—O2	2.114 (2)
Co1—N1	2.193 (2)

**Table 2:** Hydrogen-bond geometry (Å, °)

*D*—H⋯*A*	*D*—H	H⋯*A*	*D*⋯*A*	*D*—H⋯*A*
N4—H4′⋯O1	0.78 (3)	2.20 (3)	2.587 (3)	111 (2)
O2—H2′⋯N2^i^	0.81 (4)	2.11 (4)	2.899 (3)	166 (3)
O2—H2′⋯N3^i^	0.81 (4)	2.52 (4)	3.161 (3)	138 (3)

Symmetry code: (i) 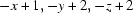.

**Fig. 1.**
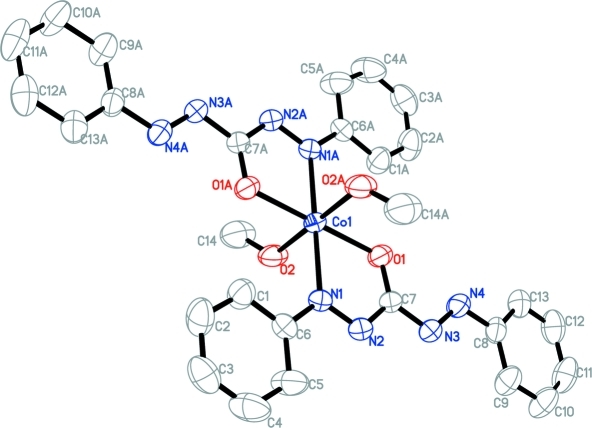
Molecular structure showing 50% probability displacement ellipsoids. H atoms are omitted for clarity.

**Fig. 2.**
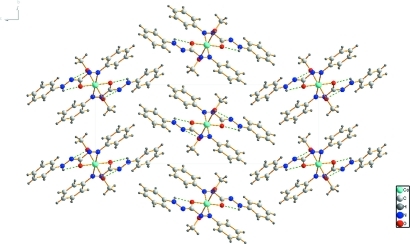
Packing diagram viewed down the a axis. Symmetry code corresponds to A:-x+2,-y+2,-z+2.

## supplementary crystallographic information

### Comment

The diphenylcarbazide is often used for analytical determination of chromium in biological materials (Yarbro *et al.* 1976). As a multidentate ligand, diphenylcarbazide chelates the metal centres by two N atoms (Feigl 1924) or coordinates with the metal ions by O atom in monodentate fashion (Shafranskii *et al.*, 1975a,b; Martynova *et al.*, 1985), whereas the examples of diphenylcarbazide complexes, in which the ligands chelated metal ions bidentately by one O atom and one N atom, were very rare (Turkington *et al.*, 1958; Deshpande *et al.*, 1988). Herein we report the synthesis and crystal structure of such diphenylcarbazide coordinated cobalt complex with Co occuping an inversion centre (Fig. 1 and Table 1). The Packing diagram of I viewed down the *a* axis (Fig. 2) reveals hydrogen bond interactions (Table 2).

### Experimental

The compound 1 was synthesized by solvothermal reaction. A mixture of diphenylcarbazide (0.0499 g, 0.2 mmol), Co(CH_3_COO)_2_.4H_2_O (0.0245 g, 0.1 mmol) and CH_3_OH / CH_3_CN (v / v = 2: 1, 2 ml) was sealed in a 5 ml glass tube and heated to 353 K for 48 h. After cooling to room temperature, purple crystals were obtained.

### Refinement

Methyl H atoms were placed in calculated positions with C—H = 0.96 Å, and torsion angles were refined, *U*_iso_(H) = 1.5 *U*_eq_(C, O). Other H atoms were placed in calculated positions with C—H = 0.93 (aromatic) or 0.803 Å (Imino) and refined in riding mode, with *U*_iso_(H) = 1.2 *U*_eq_(C).

### Crystal data

**Table:**

[Co(C_13_H_11_N_4_O)_2_(CH_4_O)_2_]	*F*(000) = 626
*M**_r_* = 601.53	*D*_x_ = 1.371 Mg m^−^^3^
Monoclinic, *P*2_1_/*c*	Mo *K*α radiation, λ = 0.71073 Å
Hall symbol: -P 2ybc	Cell parameters from 2392 reflections
*a* = 6.492 (2) Å	θ = 2.4–24.6°
*b* = 8.926 (3) Å	µ = 0.64 mm^−^^1^
*c* = 25.159 (9) Å	*T* = 296 K
β = 92.372 (6)°	Block, clear violet
*V* = 1456.7 (9) Å^3^	0.35 × 0.28 × 0.27 mm
*Z* = 2	

### Data collection

**Table:**

Bruker SMART CCD area-detector diffractometer	2564 independent reflections
Radiation source: fine-focus sealed tube	2037 reflections with *I* > 2σ(*I*)
graphite	*R*_int_ = 0.046
phi and ω scans	θ_max_ = 25.0°, θ_min_ = 1.6°
Absorption correction: multi-scan (*SADABS*; Bruker, 2001)	*h* = −7→7
*T*_min_ = 0.808, *T*_max_ = 0.847	*k* = −10→10
6990 measured reflections	*l* = −20→29

### Refinement

**Table:**

Refinement on *F*^2^	Primary atom site location: structure-invariant direct methods
Least-squares matrix: full	Secondary atom site location: difference Fourier map
*R*[*F*^2^ > 2σ(*F*^2^)] = 0.042	Hydrogen site location: inferred from neighbouring sites
*wR*(*F*^2^) = 0.117	H atoms treated by a mixture of independent and constrained refinement
*S* = 1.08	*w* = 1/[σ^2^(*F*_o_^2^) + (0.0652*P*)^2^ + 0.0401*P*] where *P* = (*F*_o_^2^ + 2*F*_c_^2^)/3
2564 reflections	(Δ/σ)_max_ < 0.001
196 parameters	Δρ_max_ = 0.40 e Å^−^^3^
0 restraints	Δρ_min_ = −0.43 e Å^−^^3^

### Special details

**Table:**

Geometry. All e.s.d.'s (except the e.s.d. in the dihedral angle between two l.s. planes) are estimated using the full covariance matrix. The cell e.s.d.'s are taken into account individually in the estimation of e.s.d.'s in distances, angles and torsion angles; correlations between e.s.d.'s in cell parameters are only used when they are defined by crystal symmetry. An approximate (isotropic) treatment of cell e.s.d.'s is used for estimating e.s.d.'s involving l.s. planes.
Refinement. Refinement of *F*^2^ against ALL reflections. The weighted *R*-factor *wR* and goodness of fit *S* are based on *F*^2^, conventional *R*-factors *R* are based on *F*, with *F* set to zero for negative *F*^2^. The threshold expression of *F*^2^ > σ(*F*^2^) is used only for calculating *R*-factors(gt) *etc*. and is not relevant to the choice of reflections for refinement. *R*-factors based on *F*^2^ are statistically about twice as large as those based on *F*, and *R*- factors based on ALL data will be even larger.

### Fractional atomic coordinates and isotropic or equivalent isotropic displacement parameters (Å^2^)

**Table:**

	*x*	*y*	*z*	*U*_iso_*/*U*_eq_	
Co1	1.0000	1.0000	1.0000	0.03524 (19)	
C1	0.8530 (5)	0.7374 (3)	1.09426 (11)	0.0528 (7)	
H1	0.9758	0.7899	1.0914	0.063*	
C2	0.8241 (6)	0.6473 (4)	1.13836 (11)	0.0640 (9)	
H2	0.9270	0.6404	1.1651	0.077*	
C3	0.6446 (6)	0.5688 (4)	1.14251 (13)	0.0642 (9)	
H3	0.6256	0.5087	1.1721	0.077*	
C4	0.4933 (5)	0.5785 (4)	1.10342 (14)	0.0720 (10)	
H4	0.3721	0.5241	1.1064	0.086*	
C5	0.5185 (5)	0.6687 (4)	1.05925 (13)	0.0634 (9)	
H5	0.4145	0.6753	1.0328	0.076*	
C6	0.7007 (4)	0.7492 (3)	1.05484 (10)	0.0382 (6)	
C7	0.6545 (4)	0.9405 (3)	0.93219 (10)	0.0367 (6)	
C8	0.4192 (4)	1.0503 (3)	0.80937 (10)	0.0437 (6)	
C9	0.2276 (5)	0.9825 (3)	0.80672 (13)	0.0584 (8)	
H9	0.1907	0.9157	0.8330	0.070*	
C10	0.0906 (6)	1.0150 (4)	0.76451 (14)	0.0710 (10)	
H10	−0.0385	0.9697	0.7627	0.085*	
C11	0.1441 (6)	1.1139 (4)	0.72520 (13)	0.0719 (10)	
H11	0.0509	1.1361	0.6972	0.086*	
C12	0.3356 (6)	1.1794 (4)	0.72765 (11)	0.0648 (9)	
H12	0.3720	1.2450	0.7009	0.078*	
C13	0.4753 (5)	1.1491 (3)	0.76934 (10)	0.0538 (7)	
H13	0.6047	1.1940	0.7707	0.065*	
C14	0.9161 (6)	1.3027 (4)	1.06416 (16)	0.0849 (12)	
H14A	1.0319	1.2726	1.0866	0.127*	
H14B	0.8090	1.3405	1.0857	0.127*	
H14C	0.9580	1.3797	1.0403	0.127*	
N1	0.7412 (3)	0.8471 (2)	1.01157 (8)	0.0359 (5)	
N2	0.6030 (3)	0.8460 (2)	0.97330 (8)	0.0391 (5)	
N3	0.5120 (3)	0.9360 (3)	0.89297 (8)	0.0411 (5)	
N4	0.5597 (4)	1.0247 (3)	0.85196 (9)	0.0463 (6)	
O1	0.8187 (3)	1.0213 (2)	0.93292 (7)	0.0438 (5)	
O2	0.8423 (3)	1.1797 (2)	1.03493 (9)	0.0570 (6)	
H4'	0.660 (5)	1.073 (3)	0.8563 (11)	0.048 (9)*	
H2'	0.720 (6)	1.167 (4)	1.0382 (13)	0.084 (12)*	

### Atomic displacement parameters (Å^2^)

**Table:**

	*U*^11^	*U*^22^	*U*^33^	*U*^12^	*U*^13^	*U*^23^
Co1	0.0269 (3)	0.0445 (3)	0.0341 (3)	−0.0019 (2)	−0.00083 (18)	−0.0004 (2)
C1	0.0589 (19)	0.0546 (17)	0.0445 (16)	−0.0123 (14)	−0.0024 (14)	0.0054 (13)
C2	0.088 (3)	0.0600 (19)	0.0428 (16)	−0.0077 (18)	−0.0077 (16)	0.0091 (14)
C3	0.086 (3)	0.0534 (18)	0.0543 (19)	−0.0007 (18)	0.0204 (18)	0.0125 (15)
C4	0.057 (2)	0.074 (2)	0.086 (3)	−0.0096 (18)	0.0160 (19)	0.029 (2)
C5	0.0419 (17)	0.076 (2)	0.073 (2)	−0.0062 (16)	0.0013 (15)	0.0258 (17)
C6	0.0402 (14)	0.0368 (13)	0.0382 (14)	0.0022 (11)	0.0080 (11)	−0.0002 (11)
C7	0.0284 (13)	0.0454 (13)	0.0362 (13)	0.0017 (11)	0.0003 (10)	−0.0023 (11)
C8	0.0474 (17)	0.0479 (15)	0.0352 (14)	0.0059 (12)	−0.0044 (12)	−0.0064 (11)
C9	0.064 (2)	0.0593 (19)	0.0508 (18)	−0.0049 (15)	−0.0145 (15)	0.0038 (14)
C10	0.066 (2)	0.078 (2)	0.066 (2)	−0.0084 (18)	−0.0298 (17)	0.0024 (18)
C11	0.087 (3)	0.071 (2)	0.0549 (19)	0.013 (2)	−0.0312 (18)	−0.0007 (18)
C12	0.093 (3)	0.0605 (19)	0.0408 (17)	0.0102 (18)	−0.0044 (16)	0.0036 (14)
C13	0.0610 (19)	0.0583 (18)	0.0420 (15)	0.0018 (15)	0.0001 (13)	−0.0031 (13)
C14	0.058 (2)	0.084 (3)	0.113 (3)	−0.0036 (19)	0.015 (2)	−0.047 (2)
N1	0.0296 (11)	0.0426 (12)	0.0355 (11)	0.0026 (9)	0.0028 (9)	−0.0024 (9)
N2	0.0313 (11)	0.0472 (13)	0.0386 (12)	0.0003 (9)	0.0013 (9)	−0.0009 (10)
N3	0.0342 (12)	0.0524 (13)	0.0365 (12)	−0.0015 (10)	−0.0017 (9)	−0.0010 (10)
N4	0.0397 (14)	0.0592 (16)	0.0394 (13)	−0.0065 (12)	−0.0059 (10)	0.0034 (11)
O1	0.0355 (10)	0.0560 (12)	0.0395 (10)	−0.0074 (9)	−0.0035 (8)	0.0049 (8)
O2	0.0295 (11)	0.0635 (13)	0.0786 (15)	−0.0035 (10)	0.0100 (10)	−0.0232 (11)

### Geometric parameters (Å, °)

**Table:**

Co1—O1^i^	2.0263 (18)	C8—C9	1.383 (4)
Co1—O1	2.0263 (18)	C8—N4	1.397 (3)
Co1—O2	2.114 (2)	C8—C13	1.398 (4)
Co1—O2^i^	2.114 (2)	C9—C10	1.387 (4)
Co1—N1^i^	2.193 (2)	C9—H9	0.9300
Co1—N1	2.193 (2)	C10—C11	1.381 (5)
C1—C6	1.375 (4)	C10—H10	0.9300
C1—C2	1.389 (4)	C11—C12	1.373 (5)
C1—H1	0.9300	C11—H11	0.9300
C2—C3	1.368 (5)	C12—C13	1.384 (4)
C2—H2	0.9300	C12—H12	0.9300
C3—C4	1.363 (5)	C13—H13	0.9300
C3—H3	0.9300	C14—O2	1.395 (4)
C4—C5	1.387 (4)	C14—H14A	0.9600
C4—H4	0.9300	C14—H14B	0.9600
C5—C6	1.392 (4)	C14—H14C	0.9600
C5—H5	0.9300	N1—N2	1.289 (3)
C6—N1	1.429 (3)	N3—N4	1.347 (3)
C7—O1	1.286 (3)	N4—H4'	0.78 (3)
C7—N3	1.325 (3)	O2—H2'	0.81 (4)
C7—N2	1.386 (3)		
			
O1^i^—Co1—O1	179.999 (1)	C9—C8—N4	121.6 (3)
O1^i^—Co1—O2	89.97 (8)	C9—C8—C13	120.1 (3)
O1—Co1—O2	90.03 (8)	N4—C8—C13	118.3 (3)
O1^i^—Co1—O2^i^	90.03 (8)	C8—C9—C10	119.5 (3)
O1—Co1—O2^i^	89.97 (8)	C8—C9—H9	120.2
O2—Co1—O2^i^	179.997 (1)	C10—C9—H9	120.2
O1^i^—Co1—N1^i^	75.34 (7)	C11—C10—C9	120.7 (3)
O1—Co1—N1^i^	104.66 (7)	C11—C10—H10	119.7
O2—Co1—N1^i^	88.27 (8)	C9—C10—H10	119.7
O2^i^—Co1—N1^i^	91.73 (8)	C12—C11—C10	119.6 (3)
O1^i^—Co1—N1	104.66 (7)	C12—C11—H11	120.2
O1—Co1—N1	75.34 (7)	C10—C11—H11	120.2
O2—Co1—N1	91.73 (8)	C11—C12—C13	121.0 (3)
O2^i^—Co1—N1	88.27 (8)	C11—C12—H12	119.5
N1^i^—Co1—N1	180.0	C13—C12—H12	119.5
C6—C1—C2	120.2 (3)	C12—C13—C8	119.1 (3)
C6—C1—H1	119.9	C12—C13—H13	120.4
C2—C1—H1	119.9	C8—C13—H13	120.4
C3—C2—C1	120.1 (3)	O2—C14—H14A	109.5
C3—C2—H2	120.0	O2—C14—H14B	109.5
C1—C2—H2	120.0	H14A—C14—H14B	109.5
C4—C3—C2	120.2 (3)	O2—C14—H14C	109.5
C4—C3—H3	119.9	H14A—C14—H14C	109.5
C2—C3—H3	119.9	H14B—C14—H14C	109.5
C3—C4—C5	120.6 (3)	N2—N1—C6	114.8 (2)
C3—C4—H4	119.7	N2—N1—Co1	114.78 (15)
C5—C4—H4	119.7	C6—N1—Co1	130.38 (16)
C4—C5—C6	119.5 (3)	N1—N2—C7	111.8 (2)
C4—C5—H5	120.3	C7—N3—N4	112.2 (2)
C6—C5—H5	120.3	N3—N4—C8	121.3 (3)
C1—C6—C5	119.4 (3)	N3—N4—H4'	116 (2)
C1—C6—N1	116.5 (2)	C8—N4—H4'	122 (2)
C5—C6—N1	124.1 (3)	C7—O1—Co1	114.33 (15)
O1—C7—N3	125.5 (2)	C14—O2—Co1	130.91 (19)
O1—C7—N2	123.8 (2)	C14—O2—H2'	112 (3)
N3—C7—N2	110.8 (2)	Co1—O2—H2'	116 (3)
			
C6—C1—C2—C3	0.6 (5)	O1—Co1—N1—C6	178.9 (2)
C1—C2—C3—C4	0.1 (5)	O2—Co1—N1—C6	−91.5 (2)
C2—C3—C4—C5	−0.5 (6)	O2^i^—Co1—N1—C6	88.5 (2)
C3—C4—C5—C6	0.4 (5)	N1^i^—Co1—N1—C6	−89 (10)
C2—C1—C6—C5	−0.8 (4)	C6—N1—N2—C7	−178.5 (2)
C2—C1—C6—N1	178.5 (3)	Co1—N1—N2—C7	1.8 (2)
C4—C5—C6—C1	0.3 (5)	O1—C7—N2—N1	−1.2 (3)
C4—C5—C6—N1	−178.9 (3)	N3—C7—N2—N1	179.1 (2)
N4—C8—C9—C10	177.9 (3)	O1—C7—N3—N4	1.5 (4)
C13—C8—C9—C10	−0.9 (5)	N2—C7—N3—N4	−178.9 (2)
C8—C9—C10—C11	0.1 (5)	C7—N3—N4—C8	−172.2 (2)
C9—C10—C11—C12	0.7 (5)	C9—C8—N4—N3	−1.8 (4)
C10—C11—C12—C13	−0.8 (5)	C13—C8—N4—N3	177.1 (3)
C11—C12—C13—C8	0.0 (5)	N3—C7—O1—Co1	179.6 (2)
C9—C8—C13—C12	0.9 (4)	N2—C7—O1—Co1	−0.1 (3)
N4—C8—C13—C12	−178.0 (3)	O1^i^—Co1—O1—C7	129 (3)
C1—C6—N1—N2	174.5 (2)	O2—Co1—O1—C7	−90.99 (18)
C5—C6—N1—N2	−6.3 (4)	O2^i^—Co1—O1—C7	89.01 (18)
C1—C6—N1—Co1	−5.9 (3)	N1^i^—Co1—O1—C7	−179.21 (17)
C5—C6—N1—Co1	173.3 (2)	N1—Co1—O1—C7	0.79 (17)
O1^i^—Co1—N1—N2	178.49 (15)	O1^i^—Co1—O2—C14	44.4 (3)
O1—Co1—N1—N2	−1.51 (15)	O1—Co1—O2—C14	−135.6 (3)
O2—Co1—N1—N2	88.07 (17)	O2^i^—Co1—O2—C14	−89 (4)
O2^i^—Co1—N1—N2	−91.93 (17)	N1^i^—Co1—O2—C14	−30.9 (3)
N1^i^—Co1—N1—N2	91 (10)	N1—Co1—O2—C14	149.1 (3)
O1^i^—Co1—N1—C6	−1.1 (2)		

Symmetry codes: (i) −*x*+2, −*y*+2, −*z*+2.

### Hydrogen-bond geometry (Å, °)

**Table:**

*D*—H···*A*	*D*—H	H···*A*	*D*···*A*	*D*—H···*A*
N4—H4'···O1	0.78 (3)	2.20 (3)	2.587 (3)	111 (2)
O2—H2'···N2^ii^	0.81 (4)	2.11 (4)	2.899 (3)	166 (3)
O2—H2'···N3^ii^	0.81 (4)	2.52 (4)	3.161 (3)	138 (3)

Symmetry codes: (ii) −*x*+1, −*y*+2, −*z*+2.

## References

[bb1] Bruker (2001). *SMART*, *SAINT* and *SADABS* Bruker AXS Inc., Madison, Wisconsin, USA.

[bb2] Cazeneuve, P. (1900*a*). *Bull. Soc. Chim. Fr.* **23**, 592–600.

[bb3] Cazeneuve, P. (1900*b*). *C. R. Hebd Seances Acad. Sci.* **130**, 1561–1563.

[bb4] Deshpande, S. G. & Jain, S. C. (1988). *Indian J. Chem. Sect. A*, **27**, 552–554.

[bb5] Feigl, F. (1924). *Ber. Wien. Akad. IIb*, **133**, 115–132.

[bb6] Martynova, T. K., Neverov, V. A., Byushkin, V. N., Shafranskii, V. N. & Malkova, T. A. (1985). *Koord. Khim.*, **11**, 132–135.

[bb7] Pankaj, & Chauhan, M. (2004). *Indian J. Chem. Sect. A Inorg. Bio-inorg. Phys. Theor. Anal. Chem.* **43**, 1206–1209.

[bb8] Shafranskii, V. N. & Mal’kova, T. A. (1975*a*). *Zh. Obshch. Khim.* **45**, 1065–1069

[bb9] Shafranskii, V. N. & Mal’kova, T. A. (1975*b*). *J. Gen. Chem. (USSR)*, **45**, 1051–1054.

[bb10] Sheldrick, G. M. (2008). *Acta Cryst.* A**64**, 112–122. 10.1107/S0108767307043930 18156677

[bb11] Sollott, G. P. & Peterson, W. R. (1969). *J. Org. Chem.* **34**, 1506–1508.

[bb12] Turkington, R. W. & Tracy, F. M. (1958). *Anal. Chem.* **30**, 1699–1701.

[bb13] Yarbro, S. & Flaschka, H. A. (1976). *Microchem* *J.* **21**, 415–423.

